# Medicinal Profile, Phytochemistry, and Pharmacological Activities of *Murraya koenigii* and Its Primary Bioactive Compounds

**DOI:** 10.3390/antiox9020101

**Published:** 2020-01-24

**Authors:** Rengasamy Balakrishnan, Dhanraj Vijayraja, Song-Hee Jo, Palanivel Ganesan, In Su-Kim, Dong-Kug Choi

**Affiliations:** 1 Department of Applied Life Sciences and Integrated Bioscience, Graduate School, Konkuk University, Chungju 27478, Korea; rmbalabio@gmail.com (R.B.); wowsong333@naver.com (S.-H.J.); 2 Department of Biochemistry, Rev. Jacob Memorial Christian College, Ambilikkai 624612, Tamilnadu, India; dvijayraja@gmail.com; 3 Department of Integrated Bio Science and Biotechnology, College of Biomedical and Health Science, Nanotechnology Research Center, Konkuk University, Chungju 27478, Korea; palanivel@kku.ac.kr

**Keywords:** *Murraya koenigii*, antioxidant, bioactive compounds, pharmacological activity, traditional medicine

## Abstract

The discovery of several revitalizing molecules that can stop or reduce the pathology of a wide range of diseases will be considered a major breakthrough of the present time. Available synthetic compounds may provoke side effects and health issues, which heightens the need for molecules from plants and other natural resources under discovery as potential methods of replacing synthetic compounds. In traditional medicinal therapies, several plant extracts and phytochemicals have been reported to impart remedial effects as better alternatives. *Murraya koenigii* (*M. koenigii*) belongs to the Rutaceae family, which is commonly used as a medicinally important herb of Indian origin in the Ayurvedic system of medicine. Previous reports have demonstrated that the leaves, roots, and bark of this plant are rich sources of carbazole alkaloids, which produce potent biological activities and pharmacological effects. These include antioxidant, antidiabetic, anti-inflammatory, antitumor, and neuroprotective activities. The present review provides insight into the major components of *M. koenigii* and their pharmacological activities against different pathological conditions. The review also emphasizes the need for more research on the molecular basis of such activity in various cellular and animal models to validate the efficacy of *M. koenigii* and its derivatives as potent therapeutic agents.

## 1. Introduction

Presently, huge numbers of people in developing countries depend on medicinal plants for healthcare, skin care, economic benefits, and cultural development. For centuries, medicinal plants have been widely used in traditional medicine in countries like India, China, Germany, Thailand, etc. [1]. The World Health Organization (WHO) projected that 80% of the population relies on traditional medicine, which is clearly elucidated by the 19.4 billion USD global revenue for herbal remedies in 2010 [2]. Moreover, the demand for traditional medicinal plants is increasing; for instance, the market for medicinal plants is expanding at an annual rate of 20% in India. Likewise, in China, 30% to 50% of the total medicinal consumption is from preparations of traditional medicine [3]. Nearly 76.7% of the citizens of Thailand have reported mainly using traditional herbal medicine for their primary healthcare [4]. Around 90% of the German population uses natural remedies for certain health issues [5]. Therefore, the medicinal plants used in traditional medical treatments are significant in both developing and industrialized countries. This is clearly demonstrated by the worldwide market for traditional medicine. This market continues to gradually increase [6]. 

*Murraya koenigii* (*M. koenigii*) (L) Spreng (Family: Rutaceae) is usually known as “curry leaves”. The tropical and subtropical regions in the world have large distributions of *M. koenigii* [7]. Among the 14 global species belonging to the genus of *Murraya*, only two, *M. koenigii* and *M. paniculate,* are available in India. *M. koenigii* is more important due to its huge spectrum of traditional medicinal properties. For centuries, this plant has been used in diverse forms and holds a place of pride in Indian Ayurvedic medicine, known as “krishnanimba” [8]. Different parts of *M. koenigii*, such as its leaves, root, bark, and fruit, are known to promote various biological activities. Aromatic bioactive constituents in the leaves of *M. koenigii* retain their flavor and other qualities, even after drying [9,10,11,12,13,14]. *M. koenigii* leaves are slightly bitter in taste, pungent in smell, and weakly acidic. They are used as antihelminthics, analgesics, digestives, and appetizers in Indian cookery [15,16]. The green leaves of *M. koenigii* are used in treating piles, inflammation, itching, fresh cuts, dysentery, bruises, and edema. The roots are purgative to some extent. They are stimulating and used for common body aches. The bark is helpful in treating snakebites [17,18,19,20]. The essential oil extracted from *M. koenigii* leaves is reported to possess anti-oxidative, hepatoprotective [21,22,23,24], antimicrobial, antifungal [25,26,27], anti-inflammatory, and nephroprotective activities in animal models [28,29,30]. The medicinal properties of *M. koenigii* have been accredited to several chemical constituents of different carbazole alkaloids and other important metabolites, like terpenoids, flavonoids, phenolics, carbohydrates, carotenoids, vitamins, and nicotinic acid from different parts of the *M. koenigii* plant.

In recent years, greater attention has been paid to the use of *M. koenigii* in traditional medicines and home remedies. On the other hand, limited studies have been conducted for evaluating the pharmacological and medicinal efficacy of *M. koenigii* in promoting health benefits and curing disease [31,32,33,34,35,36]. This review will discuss the traditional medicinal use of *M. koenigii* and its bioactive compounds, highlighting their pharmacological effects. This review aims to present a well-managed summary and possible recommendation on existing studies to provide information regarding the current reports that can direct future research. Therefore, instead of discussing a few selected studies in a specific time interval, the present review will discuss and cover previous and existing major studies on *M. koenigii* related to the topics chosen. The details, like phytochemical screening, identification, and pharmacological activities, will be systematically categorized, compared, and summarized. We hypothesized that, through all of these efforts, a good summary on pharmacological activities that could initiate future perspectives with the utmost clarity could be produced.

**Figure 1:**
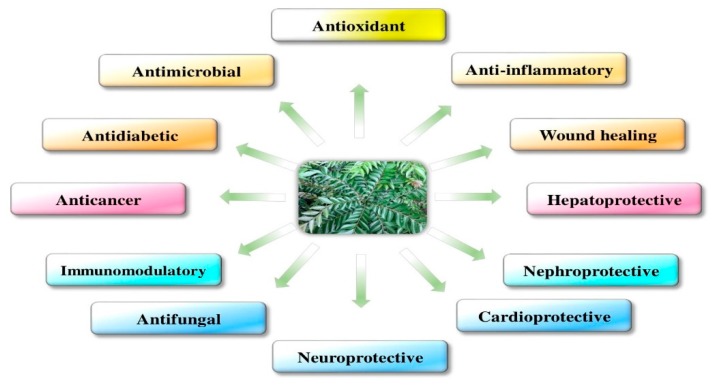
Pharmacological activities of *Murraya koenigii.*

The pharmacological activities of *M. koenigii* are discussed in detail in Figure 1.

## 2. *Murraya koenigii* (*M. koenigii*)

### 2.1. Traditional Uses of M. koenigii

Essential oils and fresh leaf powder of *M. koenigii* are useful in seasoning food items and preparing ready-to-eat foods. Owing to the higher antimicrobial activities of the essential oil from leaf extracts [37,38], this oil can also be used as perfume and flavor agents in traditional practice. Fresh curry leaves are boiled with a coconut oil mixture until they are reduced to a black residue to produce an excellent hair tonic for retaining a normal hair tone and improving hair growth. Curry leaves have a traditional use, either whole or in parts, as antidiarrheal, antifungal, blood purifying, anti-inflammatory, and anti-depressant agents [39,40,41].

### 2.2. Medicinal Uses of M. koenigii

*M. koenigii* has numerous disease remedial activities, for instance, different parts of the plant, such as the leaves, roots, and bark, can be prepared as tonics for inducing digestion and flatulence or as antiemetics [25,42]. After decoction, the leaves become bitter to the taste and are helpful in reducing fever. The juice of the root is given to manage renal pains [43]. The leaves and roots can be given as an anthelmenticku, analgesic, cure for piles, body heat reducer, and thirst quencher and are also helpful in reducing inflammation and itching. They are also useful in managing leucoderma and blood disorders. When eaten raw, the green leaves can offer a cure for dysentery, and when they are boiled in milk, the paste has good application prospects for curing poisonous bites and eruptions [44].

### 2.3. Phytochemistry of M. koenigii

A wide range of phytochemicals have been isolated from the leaves, roots, and stem bark of *M. koenigii*. *M. koenigii* extracts of leaves, root, stem bark, fruits, and seeds have yielded alkaloids, flavonoids, terpenoids, and polyphenols, as shown in Table 1. The plant leaves contain a substantial amount of proximate composition; the moisture is 63.2%, protein is 8.8%, carbohydrate is 39.4%, total nitrogen is 1.15%, fat is 6.15%, total sugars are 18.92%, starch is 14.6%, and crude fiber is 6.8%. The leaves have been reported as a significant source of several vitamins, such as vitamin A (B-carotene), with a level of 6.04 ± 0.02 mg/100 g; vitamin B3, (niacin), with a level of 2.73 ± 0.02 mg/100 g; vitamin B1 (thiamin), with a level of 0.89 ± 0.01 mg/100 g; calcium, with a level of 19.73 ± 0.02 mg/100 g; magnesium, with a level of 49.06 ± 0.02 mg/100 g; and sodium, with a level of 16.50 ± 0.21 mg/100 g. The alcohol-soluble extract has a value of 1.82%, ash has a value of 13.06%, acid-insoluble ash has a value of 1.35%, cold water (20 °C) extractive has a value of 27.33%, and maximum of hot-water-soluble extractive has a value of 33.45% [15,45]. Wide ranges of carbazole alkaloids, essential oils, terpenoids, and flavonoids have numerous beneficial roles. Table 2 summarizes the major chemical constituents of *M. koenigii* and its pharmacological activities.

### 2.4. Bioavailability Study of M. koenigii-Derived Active Constituents

An in vivo pharmacokinetic study revealed that after oral administration of the bioactive compounds at the rate of 0.1 gm/kg body weight (b.w.), the maximum systemic concentration (Cmax) of koenimbine was 1.81 ± 0.55 μM and koenidine was 2.82 ± 0.53 μM, and the time required to reach the maximum concentration was 49.8 ± 8.4 min and 240 ± 0.00 min, respectively [58]. Bhattacharya et al. demonstrated the bioavailability of mahanine—another important bioactive compound of *M. koenigii*—in mice through blood serum estimations based on high-performance liquid chromatography (HPLC) analysis. Mahanine, at a dose of 100 mg/kg of body weight, was found to take 60 min to reach the maximum concentration in blood serum [59].

## 3. Molecular Mechanism and Activities of *M. koenigii*

### 3.1. Antioxidants

Reactive oxygen species (ROS), such as singlet oxygen (O_2_), hydrogen peroxide (H_2_O_2_), the superoxide anion (O_2_•^−^), and the hydroxyl radical (•OH), are often generated as byproducts of cellular metabolic reactions and exogenous induction. These ROS create homeostatic imbalances, which lead to the generation of oxidative stress, which in turn, induces cell death and tissue injury [60]. ROS in elevated levels can damage biomolecules such as nucleic acids, proteins, and lipids [61]. Even though the antioxidant defense systems like enzymatic antioxidants and non-enzymatic antioxidants are functioning, uncontrolled ROS accumulation during the life cycle promotes the development of age-dependent diseases, like cancer, atherosclerosis, arthritis etc. [62]. Natural antioxidants from plant sources have been considered a promising therapy for the prevention and treatment of these diseases, especially neurodegenerative disorders, cardiovascular diseases, cancer, and other conditions. Various natural bioactive compounds, such as mahanine, mahanimbine, isolongifolene, koenimbine, girinimbine, isomahanine, koenoline, and O-methylmurrayamine, are present in *M. koenigii* and exhibit remarkable antioxidant properties [63,64].

The leaf extracts of *M. koenigii* have high antioxidant activities [65]. Rao et al. evaluated the antioxidant activities of water and an ethanol extract of *M. koenigii* assessed by the, α-diphenyl-β-picrylhydrazyl (DPPH) free radical scavenging assay, with quercetin as a positive control. The ethanolic extract of *M. koenigii* showed an 80% scavenging activity, which was similar to the activities exhibited by the control antioxidant compound quercetin [66]. Gupta et al. evaluated the antioxidant activities of acetone, alcohol, and aqueous extracts of *M. koenigii* by the DPPH free radical scavenging assay, with ascorbic acid as a positive control. The extracts of *M. koenigii* exhibited activities with an half-maximum effective concentration (EC_50_) value of acetone of 81.81 ± 19.92 at 4.7 µg/mL, alcohol of 79.80 ± 18.68 at 4.1 µg/mL, and aqueous extract of 62.82 ± 13.62 at 4.4 µg/mL, which was comparable to the EC_50_ value exhibited by ascorbic acid (the positive control), which was 97.13 ± 12.64 at 2.69 µg/mL [67]. Zahin et al. also evaluated the antioxidant activities of both ethyl acetate and petroleum ether fractions of *M. koenigii* through DPPH radical scavenging assay, cupric reducing antioxidant capacity (CUPRAC), and ferric reducing antioxidant power (FRAP) assays, with ascorbic acid as a positive control. The benzene fraction of *M. koenigii* was found to be the most active free radical scavenger, exhibiting an 88.3% decrease at a concentration of 100 μg/mL, followed by ethyl acetate at 79.5% and petroleum ether at 78.7%, while positive controls of ascorbic acid and butylated hydroxytoluene (BHT) at a concentration of 100 μg/mL inhibited 93.1% and 86.5% DPPH absorption, respectively. Similarly, the antioxidant activity created by reducing activity and CUPRAC assays indicated the highest reducing potential in the benzene fraction, followed by petroleum ether and ethyl acetate. The activity was greater than that of ascorbic acid and on par with that of BHT [68].

Yogesh et al. evaluated the antioxidant activity of berry extracts of *M. koenigii* by DPPH free radical scavenging activity and reducing power assays. The results indicated that an *M. koenigii* berry extract is a powerful free radical scavenger compared to known antioxidants, such as butylated hydroxytoluene, ascorbic acid, and tannic acid [69]. Tomar et al. evaluated the total antioxidant activity of acetone and petroleum ether extracts of younger and older *M. koenigii* leaves by estimating the H_2_O_2_ scavenging activity. The acetone extract of old leaves was found to have a maximum activity at 151.58%, and for young leaves in petroleum ether, the value was 72.23% [70]. Waghmare et al. evaluated the antioxidant property of fruit extracts of *M. koenigii* with DPPH free radical scavenging activity, inhibition of nitric oxide radical (NO) and thiobarbituric acid reactive substances (TBARS) activity, and reducing power assays, and •OH was also estimated, with vitamin C as a positive control. The fruit extract of *M. koenigii* exhibited antioxidant activities, and the EC_50_ value of the extracts for the DPPH assay was 2.6 mg/mL; for the NO radical, was 2.9 mg/mL; for TBARS, was 3.1 mg/mL; for the reducing power assay, was 2.7 mg/mL; and for H_2_O_2_, was 3.3 mg/mL, which were comparable to the EC_50_ value of 5 mg/mL exhibited by the vitamin C positive control [71]. The antioxidant activities exhibited by the crude extracts of *M. koenigii* were probably due to the presence of flavonoids and phenolic derivatives. The above studies revealed that various extracts of *M. koenigii* display high antioxidant activity. The studies also indicated the potential for this plant to be a natural source of strong antioxidant substances that can be used in therapy for human diseases induced by ROS.

### 3.2. Oxidative Stress

Chemical species with one or more unpaired electrons are called free radicals. In biological systems, the term “free radicals” refers to reactive oxygen species (ROS). Major ROS include O_2_•^−^, H_2_O_2_, and •OH [72]. In addition to ROS, reactive nitrogen species (RNS), including peroxynitrite (NO_3_^−^), NO, and S-nitrosothiols, also contribute to the generation of oxidative stress. Both ROS and RNS arise as intermediates in several metabolic processes and are specifically produced as part of the cellular defense against invasive pathogens. Free radicals also regulate many processes, including cellular growth, glucose metabolism, and proliferation [73].

Apart from certain beneficial effects, free radicals induce various deleterious effects. In a non-specific manner, ROS can react on significant biomolecules, which leads to deleterious effects like a loss of enzyme activity, genetic mutations and permeability alterations in the cell membrane, and RNS-induced protein *S*-nitrosylation [74]. Because DNA is constantly attacked by the free radicals, around 75,000 to 100,000 DNA damage events might occur in each cell per day. •OH is the most reactive species and interacts with all biological molecules, including the C-8 position of guanine to form 8-hydroxyguanine, which is one of the most frequently found oxidized bases in DNA [75].

An increase in the free radical concentration in the body can cause subsequent oxidative and cellular damage to lipids, proteins, RNA, and DNA [76]. The leaf extract of *M. koenigii* has recently been shown to possess potential antioxidant activity and protection against oxidative stress induced in diabetes [77]. The aqueous leaf extract of *M. koenigii* has been shown to reduce lipid peroxidation and decrease cellular damage, thereby protecting the liver from ethanol-induced toxicity [31]. Khan et al. reported the antioxidant effect and preventive effect of curry leaves in cadmium-induced oxidative stress, cardiac tissue damage, and alterations in normal cardiac functions in rats [78]. Mitra et al. reported the heavy metal chelating activity of an *M. koenigii* leaf extract. They found that there was a significant decrease in the tissue cadmium level when the rats were pre-treated with the *M. koenigii* leaf extract before cadmium administration [79].

### 3.3. Mitochondrial Dysfunction

Mitochondria are the primary source of high-energy metabolism within the cell. Mitochondria are known as the powerhouse of the living cell. Mitochondria also regulate calcium homeostasis and play a role in scavenging free radicals and controlling programmed cell death and/or the apoptosis-signaling pathway [80]. Mitochondrial damage leads to reduced adenosine triphosphate (ATP) production, increased ROS generation, impaired calcium buffering, damage to mitochondrial DNA (mtDNA), an altered mitochondrial morphology, and alterations in mitochondrial fission and fusion. All these events eventually lead to cell death [81]. It is currently believed that the majority of ROS are generated by mitochondrial complexes I and III, likely due to the release of electrons by NADH and dihydroflavine-adenine dinucleotide (FADH_2_) into the electron transport chain (ETC). Mitochondrial dysfunction is a characteristic of all chronic diseases and aging. It is characterized by a loss of efficiency in the ETC, as well as reductions in the synthesis of high-energy molecules [82]. These diseases include neurodegenerative diseases like Parkinson’s disease (PD), Alzheimer’s disease (AD), amyotrophic lateral sclerosis, multiple sclerosis, Huntington’s disease, cardiovascular diseases, auto-immune diseases, diabetes, and others [83,84].

Mitochondria, as essential organelles, have a noteworthy role in the viability of neuronal cells. Excess ROS formation is due to complex I inhibition inducing impairments in the mitochondrial membrane potential (MMP) and the pro-apoptotic members are believed to permeabilize the outer mitochondrial membrane due to the formation of oligomeric pores, which permits the release of apoptogenic molecules from the intermembrane space. Recent studies have evaluated the neuroprotective activities of isolongifolene and structurally similar compounds, such as girinimbine, murrayazoline, and O-methylmurrayamine A isolated from *M. koenigii*. By using different in vitro assays, a study reported that the above bioactive compounds exhibited the ability to restore the MMP levels and repair the mitochondrial damage [85,86,87].

### 3.4. Inflammation

Tissue injury, cell damage, infections due to pathogens, and alterations in biochemicals lead to a biological response called inflammation. In neurological disorders, the important components involved in inflammatory processes are believed to be mast cells, ependymal cells, microglia, astrocytes, and macrophages [88]. Microglia, a type of neuronal support cell acting as resident macrophages located throughout the brain by changing their morphology, actively respond to inflammation and participate in removing damaged neurons and pathogens. An ethanol extract from *M. koenigii* leaves showed significant analgesic and anti-inflammatory activity when explored using carrageenan-induced hind paw edema in albino rats [89]. Another study also confirmed the anti-inflammatory activity of an *M. koenigii* leaf extract in carrageenan-induced paw edema [90]. Additionally, the study recognized the analgesic activity of curry leaves with several experimental models. *M. koenigii* leaf extracts effectively attenuate the pain which is induced by an intraperitoneal injection of acetic acid and subplantar injection of formalin in mice, and the analgesic effect was elucidated with the writhing responses and pain responses in the late phase. Furthermore, it was reported that higher concentrations (20 and 40 mg/kg, per os (p.o.)) reduced the early-phase inflammatory responses induced by formalin [91].

Khurana et al. evaluated the in vitro and in vivo efficacy of a hydroalcoholic extract of *M. koenigii* curry leaves rich in carbazole alkaloids against lipopolysaccharide (LPS)-induced inflammation in RAW 264.7 cells. The activity of inflammatory cytokines interleukin 1 beta (IL-1β), interleukin-6 (IL-6), tumor necrosis factor (TNF-α), and p65-NFκB was significantly reduced by the hydroalcoholic extract of *M. koenigii*. In addition, the hydroalcoholic extract of *M. koenigii* reduced the expression of nitrotyrosine (NT), myeloperoxidase (MPO), IL-1β, intercellular adhesion molecule 1 (ICAM-1), and cyclooxygenase (COX-2), and increased the expression of Nrf2 [92]. Iman et al. evaluated the anti-inflammatory activity of an extract of *M. koenigii* and its bioactive compound girinimbine against lipopolysaccharide/interferon-gamma-induced RAW 264.7 cells. The girinimbine showed reduced levels of NO overproduction and pro-inflammatory cytokine levels IL-1β and TNF-α in the peritoneal fluid. These findings strongly suggest that girinimbine could act as an anti-inflammatory agent by suppressing inflammation [85]. Another study also confirmed the anti-inflammatory activity of an *M. koenigii* leaf extract. Bioactive compounds like murrayakonine A, O-methylmurrayamine A, and mukolidine were reported for their efficiency in inhibiting TNF-α and IL-6 release in LPS-induced inflammation in human peripheral blood mononuclear cells (PBMCs) [39]. The above studies have shed light on the mechanism of the anti-inflammatory activity of *M. koenigii* leaves and their active compounds, which are comparable to nonsteroidal anti-inflammatory drugs (NSAIDs).

### 3.5. Apoptosis

Apoptosis is a physiological programmed cell death mechanism which is essential for the flawless growth and development of organisms. Furthermore, it is a lively physiological course causing the self-destruction of cells that comprises lethal biochemical and morphological changes in the nucleus and cytoplasm. During cellular strain like oxidative stress and DNA damage, the process of apoptosis can arise, particularly in cells with high proliferation rates and a high expression of pro-apoptotic genes [93]. Intrinsic and extrinsic pathways regulate apoptosis, but both pathways are associated and the molecules involved in those pathways can influence one another.

Previous findings have demonstrated that an *M. koenigii* extract and its primary active compounds regulate multiple signaling pathways, including phosphatidylinositol 3 kinase (PI3K)/protein kinase B (AKT), mammalian target of rapamycin (mTOR) and mitogen-activated protein kinase (MAPK). *M. koenigii* and its primary active compounds exert complementary effects on oxidative stress and the alteration of proteins [94,95]. They are associated with mitochondrial-mediated apoptotic pathways. Murrayazoline and O-methylmurrayamine were shown to induce the downregulation of Akt/mTOR, suggesting downstream targeting of the cell survival pathway and an ability to potentiate the antitumor activity of D.L. Dexter (DLD-1) colon cancer cells; interestingly, this inhibition of the Akt/mTOR pathway could possibly activate the intrinsic apoptotic program [86]. Mahanine and isomahanine derived from *M. koenigii* leaves exert anticancer effects on oral squamous cell carcinoma cells via the induction of microtubule-associated protein 1 light chain 3, type II (LC3B-II), and cleaved caspase-3, suggesting the inhibition of autophagic flux [96]. In human leukemic cells, Mahanine was reported to induce apoptosis by interrupting signal transfer between Apo-1/Fas signaling and the Bid protein and via mitochondrial pathways in humans [59]. Recently, it was reported that girinimbine, a carbazole alkaloid isolated from *M. koenigii*, inhibited the growth of and induced apoptosis in human hepatocellular carcinoma cells (HepG2) [97]. In addition, Xin et al. reported that girinimbine inhibited ovarian cancer cell proliferation in a dose-dependent manner. It also inducted apoptosis and cell cycle arrest due to inhibition of the PI3K/AKT/mTOR and Wnt/b-catenin signaling pathways [98].

The alkaloid koenimbin found in *M. koenigii* was shown to extend pro-apoptotic activities in MCF-7 cancer cells by inhibiting glycogen synthase kinase-3 beta (GSK-3β). Koenimbin induces apoptotic cell death, phosphorylation, the accumulation of β-catenin, and the activation of nuclear factor-κB (NF-kB) in cancers. Moreover, koenimbin suppresses the expression of various anti-apoptotic genes involved in the regulation of cell proliferation and apoptosis [99]. Koenimbine has also been reported to trigger caspase activation, induce the release of cytochrome c, decrease the anti-apoptotic proteins, and increase the pro-apoptotic proteins, and all of these events lead to intrinsic apoptotic pathway activation [99]. Another study demonstrated that pyrayafoline-D and murrafoline-I isolated from *M. koenigii* could induce apoptosis in HL-60 cells. The same study also induced the loss of mitochondrial membrane potential and the subsequent activation of caspase-9/caspase-3, leading to the activation of apoptotic pathways [100].

## 4. Beneficial Pharmacological Activities of *M. koenigii* and Its Primary Active Derivatives

### 4.1. Antifungal Activity

The antifungal activity of *M. koenigii* has been reported in various studies. For example, the essential oil of the leaves was reported to possess antifungal activity [18]. The antifungal activity of the leaves of M. koenigii is due to the presence of phytochemical constituents of complex molecular structures and diverse action mechanisms, viz. alkaloids, terpenoids, flavonoids, phenolics, tannins, and saponins, which are known for their antimicrobial properties. Different investigations support the traditional use of the plant as an antifungal agent. In vitro antifungal activity may explain the use of curry leaves for the treatment of diarrhea, dysentery, and skin eruptions in folklore medicines [19]. Bioactive compounds of *M. koenigii* appreciably hold the ability of mycelial growth inhibition and thereby promote antifungal activity. The antifungal activity of *M. koenigii* against a wide range of pathogenic fungi has been studied. *Penicillium notatum, Aspergillus flavus*, *Aspergillus niger*, *Fusarium moniliforme*, *Mucor mucedo*, *Penicillium funiculosum* etc., were isolated from infected saplings and spoiled foods based on alterations of their growth characteristics, mycelial morphology, and spore morphology (Table 3) [27]. The ethanolic extract of *M. koenigii* exhibited notable effects on the hyphal morphology; namely, an increase in branching potential, which resulted in the development of short slender branches of hyphae with swollen tips. Such effects are usual for any antifungal compound. Bioactive compounds like girinimbine, murrayanine, marmesin-1′-O-beta-D’galactopyranoside, mahanine, murrayacine, mukoeic acid, murrayazolinine, girinimbilol, pyrafoline-D, and murrafoline-I are present in stem bark. Girinimbine, murrayanine, and marmesin-1′-O-beta-D’galactopyranoside have notable anti-fungal activity [20,101].

### 4.2. Antibacterial Activity

The unsystematic use of antibiotics promotes the development of multiple drug-resistant pathogenic strains of bacteria, which are very harmful, and there is a lack of proper treatment procedures for these ailments. Therefore, the need to search for new antimicrobials remains. Currently, in addition to antibiotics and chemically-synthesized drugs, curiosity for alternative medicines, such as natural or herbal medicines, is increasing. They may have fewer side effects or toxicity owing to their natural sources [102]. 

Combating microbial infections without side effects is always a tedious process. In this regard, in addition to classical antibiotics and synthetic drugs, there is an ongoing hunt for potent molecules from natural herbal medicines [102]. *M. koenigii* extracts have demonstrated antibacterial effects on a wide variety of microorganisms. Methanol and ethanol extracts of *M. koenigii* leaves were found to be effective against the bacterial strains *Escherichia coli* (*E. coli)*, *Staphylococcus*, *Streptococcus,* and *Proteus*. Hence, *M. koenigii* leaves could be efficiently used as a natural remedy in everyday meals for the prevention of several bacterial infections [103]. Pyranocarbazoles isolated from *M. koenigii* exhibited antibacterial activity on bacterial strains of *Staphylococcus aureus* and *Klebsiella pneumonia* [40]. Green synthesized silver nanoparticles (AGNPs) from *M. koenigii* exhibited therapeutic efficacy against multidrug resistant MDR bacteria [103]. *M. koenigii* essential oil showed antibiofilm activity against *Pseudomonas aeruginosa* and it was reported that *M. koenigii* essential oil treatment revealed an 80% reduction in biofilm formation by *P. aeruginosa*. Microscopic analyses confirmed the drop in biofilm formation in *Pseudomonas aeruginosa* when treated with *M. koenigii* essential oil. The presence of antibiofilm substances like spathulenol (5.85%), cinnamaldehyde (0.37%), and linalool (0.04%) was reported in gas chromatography-mass spectrometry (GCMS) studies [104]. *M. koenigii* counteraction on uropathogenic bacteria isolated from clinical samples was reported in a different study [105]. *M. koenigii* was tested for its antibiotic action against *Mycobacterium* species, which was appreciable, like first-line anti-tuberculosis drugs. *M. koenigii* half maximal inhibitory concentration ((IC_50_) 400 μg/mL) was found to be more effective against *Mycobacterium smegmatis* compared to water extracts and petroleum ether. An *M. koenigii* ethanol extract exhibited significant synergistic antibacterial activity against *Mycobacterium smegmatis* and *Mycobacterium bovis* bacillus calmette-guerin (BCG) in combination with the anti-tuberculosis drug rifampicin [106].

### 4.3. Hepatoprotective Effect

Liver diseases are a worldwide concern, and accessible medical treatments have an inadequate efficacy. Since ancient times, herbs have been used when treating various disease conditions; plant extracts and natural compounds have significant applications as hepatoprotective agents. The liver is the site of drug metabolism and the detoxification site of toxic products, and so it is the organ most exposed to xenobiotics [107]. *M. koenigii* extended hepatoprotective activity when crude aqueous extracts were investigated against ethanol-induced hepatotoxicity in experimental animals. *M. koenigii* was reported to extend a protective effect in liver impairments in chronic alcoholism and was proved to be effective in maintaining the enzymatic oxidant status [108]. Water extracts of carbazole alkaloids and tannin of *M. koenigii* were explored for their hepatoprotective activity against ethanol-induced hepatotoxicity in a HepG2 cell line model. They exhibited excellent hepatoprotective activity, maintaining the enzymatic and non-enzymatic antioxidant level at a near normal value and also maintaining the integrity of the cells [31]. An *M. koenigii* hydro-ethanolic leaf extract was reported to attenuate the CCl_4_ hepatotoxic effects in rats. *M. koenigii*-pretreated rats showed a significant decrement in activity levels of hepatic markers and also maintained the level of enzymatic antioxidants [16].

### 4.4. Immunomodulatory Activity

The immune system makes a network and regulates processes important for maintaining the health of an organism by hindering the entry and invasion of microbes. Impairments in the immune system lead to conditions from chronic inflammation to cancer [109]. In an investigation on the humoral- and cell-mediated immune response to ovalbumin, the immunomodulatory activity of a methanolic extract of *M. koenigii* leaves was evaluated using a carbon clearance test. A considerable increase in the NO production indicated the increased phagocytic activity of macrophages. The *M. koenigii* extract holds promise as an immunomodulatory agent, which acts by stimulating humoral immunity and the phagocytic function [110]. The *M. koenigii* leaf extracts were reported to have certain effects in regulating mice immunology related to oxidative stress metabolism. An *M. koenigii* leaf extract can exhibit an immunomodulatory effect through which it can regulate the oxidative stress metabolism in diabetic mice [111].

### 4.5. Nephroprotective Activity

*M. koenigii* has been used as a nephroprotective agent in a diabetic-induced rat model [9]. The *M. koenigii* leaf extract was found to be efficient in maintaining normal levels of serum creatinine, blood urea nitrogen, total serum protein, serum Na^+^, urine output, urinary creatinine, urinary urea, total urinary protein, and urinary Na^+^. Furthermore, the *M. koenigii* extract maintained the standard pattern in in vivo antioxidants, renal myeloperoxidase (MPO) activity, and histopathology of kidneys against unilateral renal ischemia reperfusion injury. Therefore, the extract of *M. koenigii* was clearly demonstrated to be useful in treating kidney disorders in rats [112]. The nephroprotective activity of *M. koenigii* was elucidated in experimental investigations, which showed decreased levels of blood urea nitrogen (BUN), serum creatinine (Cr), and lipid peroxidation (LPO). An *M. koenigii* extract is efficient against cyclophosphamide-induced nephrotoxicity, which was clearly revealed through the maintenance of high levels of glutathione (GSH) and superoxide dismutase (SOD) compared to the cyclophosphamide-treated group [28]. *M. koenigii* protective activity has been shown to induce significant dose-dependent decreases in serum urea and creatinine levels, as well as marked increases in the levels of plasma antioxidant capacity, in diabetic rats, compared to controls. More noteworthy is the histological integrity of kidneys, which showed comparable tissue regeneration induced by the aqueous extract [9].

### 4.6. Antidiabetic Activity

Most prominently in developing countries, medicinal plants play a helpful role in managing diabetes mellitus due to their cost effectiveness. Diabetes mellitus, a metabolic disorder, is becoming a serious threat to human health. During the past few years, many phytochemicals responsible for anti-diabetic effects have been isolated from plants. Alkaloids present in the leaves of *M. koenigii* have been explored and reported to have inhibitory effects on the aldose reductase enzyme, glucose utilization, and other enzyme systems for extending anti-diabetic effects [38]. *M. koenigii* was tested for the α-glucosidase inhibitory property and was found to inhibit α glycosidase. Alpha-glucosidase inhibitors are widely used in the treatment of patients with type 2 diabetes [113]. A study reported that an ethanolic extract of *M. koenigii* showed a significant reduction in blood glucose levels, and this effect of reducing blood glucose by *M. koenigii* is mediated by antioxidant properties and insulin mimetic effects. In addition, *M. koenigii* exhibited a profound antioxidant effect by reducing the malondialdehyde (MDA) level, increasing the GSH level, and significantly decreasing the homeostatic model assessment (HOMA)-insulin resistance index. On the whole, it is evident that *M. koenigii* possesses antidiabetic activity and has antioxidant effects in rats [10].

### 4.7. Anticancer Activity (In Vivo and In Vitro)

*M. koenigii* possesses potential secondary metabolites that could be developed as anticancer agents. In one study, the cytotoxic activity was evaluated for three extracts: hexane, ethyl acetate, and methanol of *M. koenigii* leaves against the HeLa cell line. The extracts were reported as being potently cytotoxic in nature in HeLa cancer cells. These results established the potential of *M. koenigii* as an anticancer agent in vitro [11]. Additional evidence for the anticancer activity of *M. koenigii* has been obtained from rodent cancer cell lines, as well as different in vivo cancer models [12,13,14,22,23,24,114,115]. In an early study, histopathological evidence showed that *M. koenigii* extract treatment generated a decline in neoplasms in the colon [85]. A methanolic extract of *M. koenigii* was reported to have the ability to reduce proliferation in breast cancer cell lines [116]. The total alkaloid extracted from *M. koenigii* leaves has been shown to have promising cytotoxic activity in breast cancer cells, with an IC_50_ of 14.4 µg/mL [117]. The anticancer activity of mahanine and isomahanine in human oral squamous cell carcinoma CLS-354 has also been reported [96].

**Figure 2:**
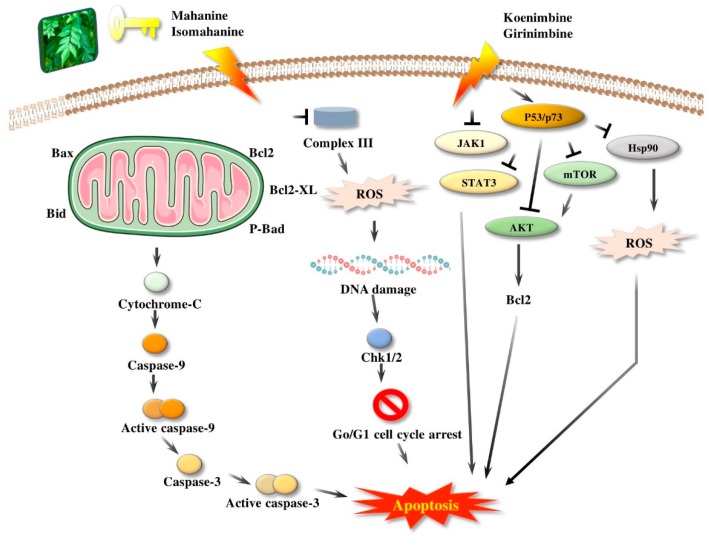
Apoptosis induced by *M. koenigii* bioactive compounds in cancer. Bcl2: B-cell lymphoma 2; Bcl2-XL: B-cell lymphoma-extra-large; P-Bad: P plasmid araB araA araD; ROS: reactive oxygen species; Chk1/2: checkpoint kinase; Go/G1: cell cycle phase; JAK1: janus kinase 1; STAT3: signal transducer and activator of transcription 3; AKT: protein kinase B (also known as AKT); mTOR: mammalian target of rapamycin; P53/p57: tumor protein; Hsp90: heat shock protein.

Girinimbine, another *M. koenigii*-derived carbazole alkaloid, showed growth inhibitory activity in human hepatocellular carcinoma and lung cancer cells in vitro [118]. Rutin, quercetin, kaempferol, and apigenin, present in leaf extracts of *M. koenigii*, showed the dose-dependent inhibition of endogenous 26S proteasome activity in MDA-MB-231 cells [52]. Therefore, *M. koenigii* contains remarkable anticancer compounds, especially mahanine, which has been reported to show anticancer activity targeting different signaling pathways [46]. Girinimbine, a carbazole alkaloid, has been found to have a good role in total leukocyte migration and result in an appreciable reduction in pro-inflammatory cytokine levels. The various activities of *M. koenigii* against different cancer cell lines are shown in Figure 2.

### 4.8. Neuroprotective Activity

**Figure 3:**
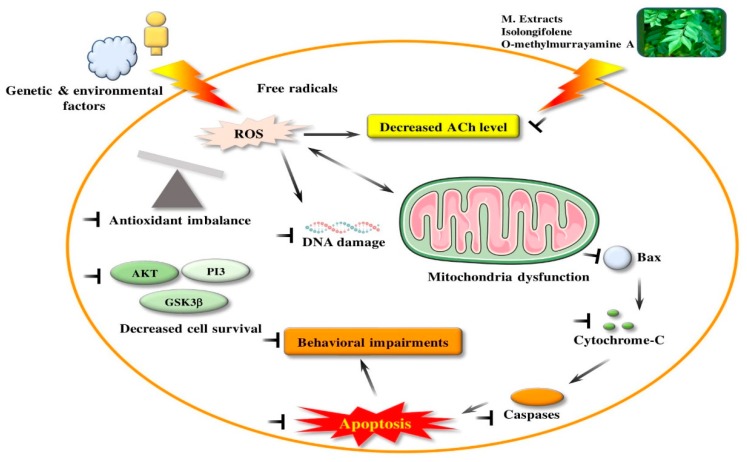
Neuroprotective effect in in vitro and in vivo studies produced by bioactive compounds from *M. koenigii.* PI3: phosphatidylinositol 3 kinase; GSK3β: Glycogen synthase kinase 3 beta; Ach: Acetylcholine; Bax: Bcl2-Associated X protein.

Supplementation with *M. koenigii* leaf extracts has been reported in the management of a wide spectrum of neurodegenerative diseases, like AD, PD, and others [30,33,34,35,41]. *M. koenigii* promotes neuroprotective potential against orofacial dyskinesia induced by resperine. Additionally, it stabilizes the levels of protective antioxidant enzymes like SOD, catalase (CAT), and GSH, and inhibits LPO in the forebrain regions of reserpine-treated animals. Furthermore, it has been shown to significantly inhibit reserpine-induced abnormalities in behavior. Similarly, treatment with *M. koenigii* significantly restored the levels of protective antioxidant enzymes (that is, SOD, CAT, and GSH) and inhibited LPO in the forebrain region when compared with reserpine, and it also inhibited catalepsy induced by haloperidol [36]. Isolongifolene (ILF), a tricyclic sesquiterpene of *M. koenigii*, has been reported to render neuroprotective effects against rotenone-induced mitochondrial dysfunction, oxidative stress, and apoptosis in a cellular model. Cytotoxicity, oxidative stress, and mitochondrial dysfunction were also attenuated by ILF in SH-SY5Y cells, which down-regulated Bax and caspases-3, -6, -8, and -9 expression, and up-regulated Bcl-2 expression. IFL was proved to regulate p-P13K, p-AKT, and p-GSK-3 beta expressions [87]. Preclinical studies have reported that *M. koenigii* leaves could enhance memory in rats [119]. The possible neuroprotective potential of amethanolic extract of *M. koenigii* leaves was exhibited in a two-vessel occlusion (2VO) rat model of partial global cerebral ischaemia. The Morris water maze test was implemented to assess the rats’ cognitive function postoperatively. Brain samples were histopathologically examined for viable neurons within the CA1 hippocampal region. Test findings showed that *M. koenigii* leaves positively improved memory and learning impairments. *M. koenigii* leaf extracts modestly improved memory in rats with chronic partial global cerebral ischemia [120]. The various activities of *M. koenigii* against neurotoxicity are shown in Figure 3.

### 4.9. Radioprotective and Chemoprotective Activity

A methanolic extract of *M. koenigii* was demonstrated to render protection in chromosomal damage against radiation and cyclophasphamide in vivo. Radiation leads to a rise in all types of aberrations, like the fragmentation of chromatids and breakages in chromosomes, rings, and dicentrics. Treatment with a methanolic extract of *M. koenigii* before radiation significantly reduced the aberrations. *M. koenigii* can significantly exert bone marrow protection against radiation and cyclophasphamide [121].

### 4.10. Wound Healing Effect

Wound healing is a complex and multifactor process involving numerous biochemical and cellular processes which helps in the restoration of functional and anatomical continuity. *M. koenigii* leaves extend wound healing in male albino rats through significantly increased wound contraction and reduced epithelialization, supporting the collagen synthesis which was evident in histopathological studies [122].

## 5. Conclusions

The current review summarizes the medicinal uses, phytochemistry, and pharmacological properties of *M. koenigii*. *M. koenigii* is a source of several bioactive compounds, including alkaloids, polyphenol, terpenoids, and flavonoids. *M. koenigii* and its derivatives appear to exhibit appreciable pharmacological activities, like anticarcinogenic, proapoptotic, antiangiogenic, antimetastatic, immunomodulatory, and antioxidant properties. The molecular mechanisms underlying these activities of *M. koenigii* and its derivatives are due to their diversified role in combinations of cell signaling pathways at multiple levels in various diseases. *M. koenigii* and its derivatives mitigate oxidative stress, neurotoxicity, neuroinflammation, neuronal loss, and cognitive dysfunctions. However, like other polyphenols, to a certain extent, *M. koenigii* activities are limited by its bioavailability and in such conditions, enhancement of the efficiency should be conducted. Therefore, future studies need to include more experimental studies on bioavailability and efficiency enhancement in clinical investigations.

## Figures and Tables

**Table 1:** Phytochemical compounds identified from *M. koenigii.*

Compound	Molecular Formula	Plant Part	References
**Alkaloids**			
Mahanine	C_23_H_25_NO_2_	Leaves, stem bark, and seeds	[45,46,47,48,49]
Mahanimbine	C_23_H_25_NO	Leaves, roots, seeds, and fruits	[47,48,49]
Murrayanol	C_24_H_29_NO_2_	Leaves, roots, and fruits	[47,48,49]
Koenimbine	C_19_H_19_NO_2_	Leaves, seeds, and fruits	[46,47,48,49]
*O*-Methylmurrayamine A	C_19_H_20_NO_2_	Leaves	[45,46,47,48]
Koenigicine	C_20_H_21_NO_3_	Leaves	[45,46,47,48]
Koenigine	C_19_H_19_NO_3_	Leaves and stem bark	[47,48,49]
Murrayone (Coumarine)	C_15_H_14_O_4_	Leaves	[45,46,47,48]
Mahanimbicine	C_23_H_25_NO	Leaves	[45,46,47,48]
Bicyclomahanimbicine	C_23_H_25_NO	Leaves	[45,46,47,48]
Phebalosin	C_15_H_14_O_4_	Leaves	[45,46,47,48]
Isomahanimbine	C_23_H_25_NO	Leaves and roots	[45,46,48]
Koenimbidine	C_20_H_21_NO_3_	Leaves and roots	[45,46,48]
Euchrestine B	C_24_H_29_NO_2_	Leaves	[45,46]
Bismurrayafoline E	C_48_H_56_N_2_O_4_	Leaves	[45,46]
Isomahanine	C_23_H_25_NO_2_	Leaves, seeds, and fruits	[45,46,49]
Mahanimbinine	C_23_H_27_NO_2_	Leaves and seeds	[45,46,49]
Girinimbilol	C_18_H_19_NO	Leaves	[45,46]
Pyrayafoline-d	C_23_H_25_NO_2_	Leaves and stem bark	[45,46,49]
Glycozoline	C_14_H_13_NO	Leaves	[45,46]
Cyclomahanimbine	C_23_H_25_NO	Leaves	[45,46]
Isomurrayazoline	C_23_H_25_NO	Leaves	[45,46]
Mahanimboline	C_23_H_25_NO_2_	Leaves	[49]
Mukonicine	C_20_H_21_NO_3_	Leaves	[49]
Isolongifolene	C_15_H_24_	Leaves	[49]
Mukonal	C_13_H_9_NO_2_	Stems	[49]
Mukeic acid	C_14_H_11_NO_3_	Stems	[49]
9-Carbethoxy-3-methyl carbazole	C_16_H_15_NO_2_	Roots and stems	[49]
9-Formyl-3-methyl carbazole	C_14_H_11_NO	Roots and stems	[49]
Murrayazolinol	C_23_H_25_NO_2_	Stems bark	[49,50]
Mahanimbinol	C_23_H_27_NO	Stems bark	[49,50]
Mukoeic acid	C_14_H_11_NO_3_	Stem bark	[49,50]
Osthol	C_15_H_16_O_3_	Stem bark	[49,50]
Umbelliferone	C_9_H_6_O_3_	Stem bark	[49,50]
Murrayanine	C_14_H_11_NO_2_	Stem bark	[49,50]
Mukoenine-A	C_18_H_19_NO	Roots and stem bark	[49,50]
Mukoenine-B	C_23_H_25_NO_2_	Roots and stem bark	[49,50]
Mukoline	C_14_H_13_NO_2_	Roots	[49,50]
Mukolidine	C_14_H_11_NO_2_	Roots and stem bark	[49,50]
(M)-murrastifoline-F	C_28_H_24_N_2_O_2_	Roots and stem bark	[49,50]
3-Methyl-9H-carbazole-9-carbaldehyde	C_14_H_11_NO	Roots	[49,50]
Bismahanine	C_46_H_48_N_2_O_4_	Roots and stem bark	[49,50]
Bikoeniquinone A	C_27_H_20_N_2_O_3_	Roots and stem bark	[49,50]
Bismurrayaquinone	C_26_H_16_N_2_O_4_	Roots and stem bark	[49,50]
3-Methylcarbazole	C_13_H_11_N	Roots	[49]
Murrayafoline A	C_14_H_13_NO	Roots	[49]
Murrayakonine A	C_37_H_36_N_2_O_2_	Leaves and stems	[39]
Murrayakonine B	C_23_H_23_NO_2_	Leaves and stems	[39]
Murrayakonine C	C_24_H_25_NO_3_	Leaves and stems	[39]
Murrayakonine D	C_23_H_25_NO_2_	Leaves and stems	[39]
Girinimbine	C_18_H_17_NO	Roots, stem bark, and seeds	[49,51]
Murrayacine	C_18_H_15_NO_2_	Stem and bark	[49,51]
Murrayazoline	C_23_H_25_NO	Stem and bark	[49,51]
**Flavonoids**			
Quercetin	C_15_H_10_O_7_	Leaves	[52]
Apigenin	C_15_H_10_O_5_	Leaves	[52]
Kaempferol	C_15_H_10_O_6_	Leaves	[52]
Rutin	C_27_H_30_O_16_	Leaves	[52]
Catechin	C_15_H_14_O_6_	Leaves	[52]
Myricetin	C_15_H_10_O_8_	Leaves	[52]
4-*O*-β-d-Rutinosyl-3-methoxyphenyl-1-propanone	C_22_H_32_O_12_	Leaves	[53]
1-*O*-β-d-Rutinosyl-2(*R*)-ethyl-1-pentanol	C_19_H_36_O_10_	Leaves	[53]
8-Phenylethyl-*O*-β-d-rutinoside	C_20_H_30_O_10_	Leaves	[54]
**Terpenoids**			
Blumenol A	C_13_H_20_O_3_	Leaves	[53]
Icariside B1	C_19_H_30_O_8_	Leaves	[53]
Loliolide	C_11_H_16_O_3_	Leaves	[53]
Blumenol A	C_13_H_20_O_3_	Leaves	[53]
Icariside B1	C_19_H_30_O_8_	Leaves	[53]
(−)-Epiloliolide	C_11_H_16_O_3_	Leaves	[55]
(−)-α-pinene	C_10_H_16_	Leaves	[55]
(−)-β-pinene	C_10_H_16_	Leaves	[55]
(+)-β-pinene	C_10_H_16_	Leaves	[55]
(+)-sabinene	C_10_H_16_	Leaves	[55]
Squalene	C_30_H_50_	Leaves and bark	[56]
β-sitosterol	C_29_H_50_O	Leaves and bark	[56,57]
**Polyphenols**			
Selin-11-en-4α-ol	C_15_H_26_O	Leaves and bark	[56]
2-hydroxy-4-methoxy-3,6-dimethylbenzoic acid	C_10_H_12_O_4_	Bark	[56]

**Table 2:** The major bioactive compounds of *M. koenigii* and its pharmacological activities.

Serial No.	Constituent	Constituent Structure	Activity
1	Mahanine	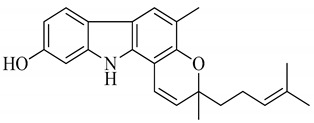	Cytotoxicity, anti-microbial, and anti-cancer
2	Mahanimbine	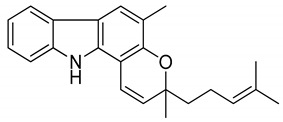	Cytotoxicity, anti-oxidant, anti-microbial, anti-diabetic, and hyperlipidemic
3	Isomahanine	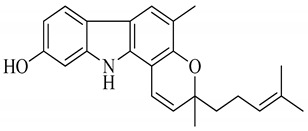	Cytotoxicity, anti-oxidant, anti-microbial, anti-diabetic, and hyperlipidemic
4	koenimbine	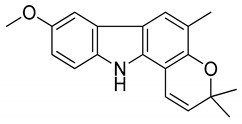	Cytotoxicity and anti-diarrhea
5	Girinimbine	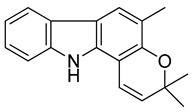	Anti-tumor
6	Isolongifolene	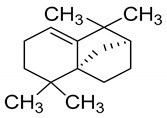	Anti-oxidant and neuroprotective
7	Pyrayafoline D	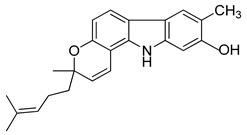	Anti-cancer and anti-bacterial
8	Murrayafoline	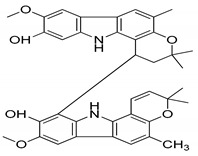	Cytotoxicity and anti-inflammatory
9	Murrayazoline	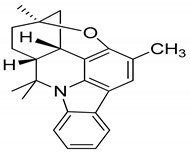	Cytotoxicity and anti-tumor
10	Koenoline	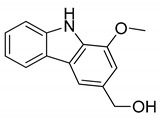	Cytotoxicity
11	9-formyl-3-methyl carbazole	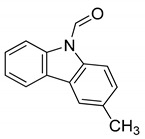	Anti-oxidant
12	*O*-Methylmurrayamine	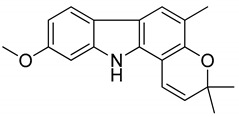	Anti-oxidant and neuroprotective
13	Koenine	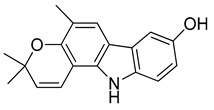	Anti-oxidant
14	Koenigine	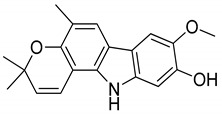	Anti-oxidant
15	Mukonicine	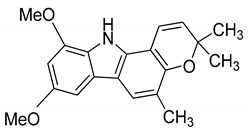	Anti-oxidant
16	Mahanimbinine	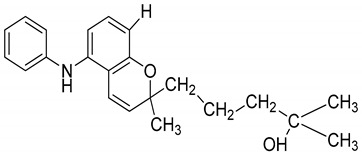	Anti-oxidant, anti-microbial, anti-diabetic, and hyperlipidemic
17	Murrayacinine	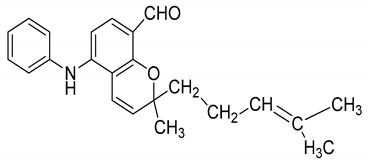	Anti-oxidant, anti-microbial, anti-diabetic, and hyperlipidemic
18	Mahanimboline	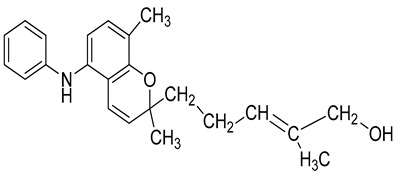	Cytotoxicity, anti-oxidant, anti-microbial, anti-diabetic, and hyperlipidemic
19	Mukoeic acid	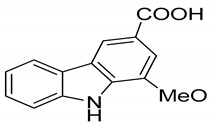	Anti-oxidant
20	Murrayanine	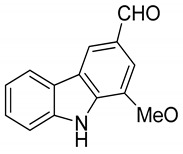	Anti-oxidant

**Table 3:** An overview of the pharmacological activities of *M. koenigii* and its primary bioactive compounds.

Pharmacological Activities	Plant Parts	Extract	Bioactive Compounds	Model	Main Finding	Reference
**In vitro studies**						
Antifungal	Leaves	Essential oil	−	Disc diffusion method	Essential oil extracted from *M. koenigii* exhibited activities with MIC in the range of 25.5 to 75 μg/mL against pathogenic fungi *A. niger*, *F. moniliforme, P. notatum, M. mucedo*, and *P. funiculosum*	[27]
Antibacterial	Leaves	Solvent-free microwave extraction	−	Soy agar	Minimum inhibitory concentrations (MIC) of solvent-free microwave extraction (SFME) and hydro-distilled oil from *M. koenigii* with values of 400 and 600 μg/mL against *L. innocua* SFME-essential oil at 300 μg/mL provided 92% inhibition, indicating its antibacterial potential	[37]
Antibacterial	Leaves	Methanol	Koenine, koenigine, and mahanine	Broth micro-dilution assay	Koenine, koenigine, and mahanine extracted from *M. koenigii* exhibited activities with MIC values of 3.12–12.5 µg/mL against bacterial strains *S. aureus* and *K. pneumonia*	[40]
Antibacterial	Leaves	Aqueous	−	Agar diffusion assay	*M. koenigii*-AGNPs exhibited inhibitory activity against *E. coli* and *S. aureus*, with a value of 16 mm for *M. koenigii*-AgNPs and 15 mm for AgNO_3_ solution at 100 µg/well	[18]
Antibacterial	Leaves	Essential oil	−	Microtiter assay	Essential oil extracts of *M. koenigii* treatment resulted in a reduction of biofilm formation in *P. aeruginosa* PAO1. *M. koenigii* essential oil may effectively control *Pseudomonas* biofilms in indwelling medical device	[19]
Antibacterial	Leaves	Petroleum ether, ethanol, and water	−	Colony-forming unit (CFU) assay	Ethanol extracts of *M. koenigii* exhibited activity half maximal inhibitory concentration ((IC_50_) of 400 μg/mL) against the mycobacterium smegmatis compared to petroleum ether and water extracts	[20]
Hepatoprotective	Leaves	Aqueous	−	Hep G2 cell line	*M. koenigii* leaves preventing alcohol-induced cellular damage	[31]
Antioxidant	Leaves	Ethanol	−	DPPH free radical scavenging assay	exhibited activities with IC_50_ values of 21.4–49.5 µg/mL	[21]
Antioxidant	Leaves	Aqueous	−	TBARS, CAT, SOD, and glutathione (GSH) assay	Carbazole alkaloids from *M. koenigii* extract exhibited activity with IC_50_ values of 120 μg/mL in an ethanol-induced hepatotoxicity in vitro model	[31]
Antioxidant	Leaves	Aqueous/zinc oxide nanoparticles	−	DPPH free radical scavenging assay	Zinc oxide nanoparticle-synthesized *M. koenigii* extract exhibited activity with an IC_50_ value of 36.46 μg/mL	[64]
Antioxidant	Leaves	Aqueous/zinc oxide nanoparticles	−	ABTS radical scavenging assay	Zinc oxide nanoparticle-synthesized *M. koenigii* extract exhibited activity with an IC_50_ value of 11.55 μg/mL	[64]
Antioxidant	Leaves	Aqueous/zinc oxide nanoparticles	−	Superoxide assay	Zinc oxide nanoparticle-synthesized *M. koenigii* extract exhibited activity with an IC_50_ value of 11.47 μg/mL	[64]
Antioxidant	Leaves	Aqueous/zinc oxide nanoparticles	−	H_2_O_2_ Assay	Zinc oxide nanoparticle-synthesized *M. koenigii* extract exhibited activity with an IC_50_ value of 54.06 μg/mL	[64]
Antioxidant	Leaves	Ethanoic	−	DPPH free radical scavenging assay	The ethanoic extract of *M. koenigii* showed an 80% scavenging activity, which was similar to the activities exhibited by the control antioxidant compound quercetin	[66]
Antioxidant	Leaves	Aqueous, alcohol, and acetone	−	DPPH free radical scavenging assay	The extracts of *M. koenigii* exhibited activities with an EC_50_ value of acetone of 4.7 µg/mL, alcohol of 4.1 µg/mL, and aqueous of 4.4 µg/mL, which were comparable to the EC_50_ value of 2.6 µg/mL exhibited by ascorbic acid, which was the positive control	[67]
Antioxidant	Leaves	Petroleum ether and ethyl acetate	−	Cupric-reducing antioxidant capacity	CUPRAC assays indicated the highest reducing potential in the benzene fraction, followed by petroleum ether and ethyl acetate	[68]
Antioxidant	Leaves	Benzene, ethyl acetate, acetone, methanol, and ethanol	−	DPPH free radical scavenging assay	Results showed that for 100 µg/mL, the benzene fraction extracted from *M. koenigii* showed 88.3% free radical scavenging activity, followed by ethyl acetate (79.5%), petrol ether (78.7%), acetone (66.1%), methanol (50.7%), and ethanol (53.0%) fractions, respectively, with the positive control being ascorbic acid (93.1%)	[69]
Antioxidant	Fruits	Aqueous	−	DPPH free radical scavenging assay	Fruit extracted from *M. koenigii* exhibited activities with an EC_50_ value of 2.6 mg/mL	[71]
Cytotoxicity	Stem bark and roots	Hexane, chloroform, and methanol	Girinimbine	MTT assay	Girinimbine was shown to significantly inhibit the proliferation of HT-29 cells with an IC_50_ value of 4.79 ± 0.74 μg/mL.	[85]
Cytotoxicity	Leaves	Ethanol	Murrayazoline and O-methylmurrayamine A	MTT assay	Murrayazoline and O-methylmurrayamine A exhibited activities with IC_50_ values of 5.7 and 17.9 mM in both HEK-293 and HaCaT cell lines, respectively	[86]
Cytotoxicity	−	−	Isolongifolene	MTT assay	Isolongifolene exhibited activities at 10 µM, showing a 90% viability in SH-SY5Y cells	[87]
Cytotoxicity	Leaves	Methanol	−	MTT assay	*M. koenigii* methanolic extract exhibited activities with IC_50_ values >400 µg/mL in the CLS-354 cell line	[96]
Cytotoxicity	Leaves	Ethanol	−	MTT assay	*M. koenigii* ethanolic extract exhibited activities with an IC_50_ value of 20 µg/mL in the mouse macrophage RAW 264.7 cell line	[20]
Cytotoxicity	Leaves	Hexane, ethyl acetate, and methanol	−	MTT assay	Three extracts of *M. koenigii* exhibited were very active, with values of <1 μg/mL to 2.25 μg/mL, and were thus proved to be potent cytotoxic activity agents against HeLa cancer cells	[11]
Anti-inflammatory	Stems	Methanol	Murrayakonine A, murrayanine, and O-methylmurrayamine-A	Human peripheral blood mononuclear cells	In vitro experiments showed murrayakonine A (IC_50_ 10 µM), murrayanine (IC_50_ 9.4 µM), and O-methylmurrayamine-A (IC_50_ 7 µM) against TNF-α, and murrayanine (IC_50_ 8.4 µM) and methylmurrayamine-A (IC_50_ 8.4 µM) against IL-6, respectively	[39]
Anticancer (Colon)	Leaves	Ethanol	O-methylmurrayamine 5.7–17.9 µM	MCF-7 cells	O-methylmurrayamine A exhibited anti-colon cancer activity through downregulation of the Akt/mTOR survival pathway and activation of the intrinsic pathway of apoptosis	[86]
Anticancer (Oral)	Leaves	Methanol	Mahanine 15 μM	CLS-354 cells	Mahanine increased the expression of LC3B-II, cleaved caspase-3 proteins, and the inhibition of autophagic flux	[96]
Anticancer (Ovarian)	Stem bark	Methanol	Girinimbine 10 µM	Ovarian cancer cell line SKOV3/ SV40	Girinimbine was found to be mainly due to the induction of apoptosis and cell cycle arrest due to the inhibition of the PI3K/AKT/mTOR and Wnt/b-catenin signaling pathways	[98]
Anticancer (Breast)	Leaves	Aqueous acetone	Koenimbin 4.89 μg/mL	MCF7 breast cancer stem cells	Koenimbin induced apoptosis in MCF7 cells that was mediated by cell death and regulated the mitochondrial membrane potential by downregulating Bcl2 and upregulating Bax, due to cytochrome *c* release from the mitochondria to the cytosol, and significantly downregulated the Wnt/β-catenin self-renewal pathway	[98]
Anticancer (Prostate)	Leaves	Aqueous acetone	Koenimbin 3.73 μg/mL	Prostate cancer stem cells	Koenimbin induced apoptosis through the intrinsic signaling pathway and suppression of the translocation of cytoplasmic NF-κB into the nucleus, in addition to displaying potential for targeting PCSCs, as affirmed by the prostasphere formation and Aldefluor assay	[99]
Anticancer	Leaves	Methanol	Mahanine 7.5 μM	Glioma HS 683 cells	Mahanine inhibited the cell migration and invasion and inhibited cell growth was simultaneous with the suppression of p-PI3K, p-AKT, and p-mTOR	[96]
Anticancer (Liver)	Leaves	Methanol	Mahanine 25 μM	HepG2, HuCCT1, and KKU-100 cells	Mahanine showed potent cytotoxicity, with increased expression levels of MITF balance between the cellular stresses	[13]
Anticancer (Cervical)	Leaves	Methanol	Mahanine 8.6 μM	HeLa (HPV-18) and SiHa (HPV-16) cell line	Mahanine and cisplatin synergistically displayed growth inhibitory activity in cervical cancer, the inhibition of STAT3 activation, cell migration, and induced apoptosis	[14]
Anticancer (Lung)	Leaves	Methanol	Mahanine 15 μM	NSCLC cancer cell line A549	Mahanine induced the impairment of mTORC2 through rictor inhibition and the destruction of NSCLC cancer cells	[22]
Anticancer (Colon)	Leaves	Methanol	Mahanine 0–30 μM	HCT116, HCT116, SW480, and Vero	Mahanine synergistically activated the two tumor suppressors PTEN and p53/p73 and can potentially be used in combination therapy with 5-FU for the treatment of colon carcinoma	[23]
Anticancer (prostate)	Leaves	Methanol	Mahanine 10 μM	PC3 and LNCaP cell line	Mahanine selectively degraded DNMT1 and DNM T3B via the ubiquitin-proteasomal pathway in a dose-dependent manner upon the inactivation of Akt signaling	[24]
Neuroprotective	Leaves	Methanol	Isolongifolene 10 µM	SH-SY5Y cells	Isolongifolene was effectively attenuated in oxidative stress, mitochondrial dysfunction, and apoptosis	[87]
Neuroprotective	Leaves	Methanol	O-methylmurrayamine A	PC12 cells	O-methylmurrayamine A possibly protects against DNA damage, apoptosis, and high levels of cell viability	[35]
**In vivo studies**						
Antioxidant	Leaves	Aqueous	−	Male albino Wistar rat	The oral administration of an *M. koenigii* leaf extract resulted in a significant reduction in the level of TBARS in both the plasma (3.64 ± 0.13) and pancreas (53.40 ± 2.13) of diabetic rats	[61]
Antioxidant	Leaves	Aqueous	−	Male albino Wistar rat	The oral administration of an *M. koenigii* leaf extract resulted in a significant increase in the level of GSH in both the plasma (24.16 ± 1.30) and pancreas (19.52 ± 1.09) of diabetic rats	[77]
Antioxidant	Leaves	Aqueous	−	Male albino Wistar rat	The oral administration of an *M. koenigii* leaf extract significantly restored the activity of SOD in the pancreas (3.69 ± 0.15) of diabetic rats	[77]
Antioxidant	Leaves	Aqueous	−	Male albino Wistar rat	The oral administration of an *M. koenigii* leaf extract significantly restored the activity of CAT in the pancreas (12.94 ± 0.54) of diabetic rats	[77]
Antioxidant	Leaves	Aqueous	−	Male albino Wistar rat	The oral administration of an *M. koenigii* leaf extract significantly restored the activity of GPx in the pancreas (5.86 ± 0.22) of diabetic rats	[67]
Antioxidant	Leaves	Ethanol	−	Sprague Dawley rats	For 200 and 400 µg/mL b.w, the *M. koenigii* extract showed 80% inhibited free radical generation and 75% restored GSH levels	[10]
Antioxidant	Leaves	Water	−	Male albino Wistar rat	Extract exhibited the potential to reduce lipid peroxidation activity in the liver (2.44 ± 0.029) and kidney (2.34 ± 0.09) in potassium dichromate-induced Wistar rats	[38]
Anti-inflammatory	Stem bark and roots	Hexane, chloroform, and methanol	Girinimbine	Adult zebrafish	Girinimbine treatment significantly suppressed the IL-1β and TNF-α levels induced by peritoneal fluid mice	[85]
Anti-inflammatory	Leaves	Ethanol	−	Sprague Dawley rats	Oral administration of an *M. koenigii* extract showed the reduced formation of oedema, with values of 43.28%, 59.67%, and 62.29% induced by carrageenan, histamine, and serotonin in rats	[30]
Hepatoprotective	Leaves	Hydro-ethanolic	−	Male Wistar rats	*M. koenigii* leaves significantly decreased CCl4 -induced hepatotoxic in a time- and dose-dependent manner	[16]
Nephroprotective	Leaves	Aqueous	−	Male Wistar rats	*M. koenigii* extract treatment significantly decreased the renal functional markers, like the blood urea nitrogen and creatinine level	[28]
Anti-Diabetic	Leaves	Ethanol	−	Swiss albino mice	*M. koenigii* possesses antidiabetic activity and has antioxidant effects on STZ-NA-induced diabetes mellitus and particularly significantly decreased the HOMA-IR index	[10]
Anticancer (Colon)	Stem bark and roots	Hexane, chloroform, and methanol	Girinimbine 1.5–100 µg/mL	Zebrafish and Male ICR mice	Girinimbine, supplementation specifically, resulted in the induction of apoptosis, the inhibition of inflammation, and a significant increase in cell numbers in the G0/G1 phase	[85]
Anticancer (Breast)	Leaves	Aqueous	−	Female BALB/c mice	*M. koenigii* aqueous extract has potential for cytotoxicity, anti-inflammatory, and immunomodulatory effects and delays rather than inhibits tumor formation	[12]
Neuroprotective	Leaves	Methanol	−	Male albino mice	*M. koenigii* is effective in attenuating memory impairment and oxidative stress and prevents abnormal oral movements	[36]
Neuroprotective	Leaves	Ethanol	−	Swiss albino mice	*M. koenigii* supplementation resulted in an improvement of acetylcholine (ACh) and reduction in acetylcholinesterase (AChE). In addition, a significant elevation of serum biomarkers, and decline in creatinine, total cholesterol, urea nitrogen, and glucose levels, ameliorated the hepatic and renal functions in the normal ageing process	[30]
Neuroprotective	Leaves	Ethanol	−	Male swiss albino mice	*M. koenigii* leaves elevated the acetylcholine level in the brain and ultimately improved memory impairment. In vitro, it showed BACE1 inhibition and was found to be a non-competitive inhibitor	[33]
Neuroprotective	Leaves	Methanol	Isolongifolene 10 mg/kg b.w.	Male albino Wistar rat	Isolongifolene effectively attenuated behavioral impairment and oxidative stress, acting as an antiaging agent	[34]
Anti-anxiety and anti-depressant	Leaves	Aqueous	−	Swiss albino mice	*M. koenigii* aqueous leaf extract reduced the despair behavior in experimental animal models, suggesting an anti-depressant-like activity and also reduced spontaneous locomotor activity	[41]
